# Depression among older adults in an urban slum of Raipur city – a community based cross-sectional study

**DOI:** 10.1186/s12877-023-04402-2

**Published:** 2023-11-02

**Authors:** Mohan Kumar, Manisha Ruikar, V. L. Surya

**Affiliations:** 1 Department of Community Medicine, KMCH Institute of Health Sciences and Research, Coimbatore, Tamil Nadu 641004 India; 2 Foundation for People-Centric Health Systems, New Delhi, India; 3 Department of Community and Family Medicine, All India Institute of Medical Sciences, Raipur, Chhattisgarh India; 4 Department of Microbiology, Coimbatore Medical College, Coimbatore, Tamil Nadu India

**Keywords:** Depression, Older adults, Geriatric, Prevalence, Determinants, India

## Abstract

**Background:**

Older adults are at risk of chronic, silent depressive changes and the vulnerability of older adults in urban slums of India is rarely exposed. The objective of this study was to estimate the prevalence of depression among the older adults in the urban slums of India and to study the factors associated with it.

**Methods:**

This was a community based analytical cross-sectional study conducted in Urban Field Practice Area of a tertiary care teaching hospital in Chhattisgarh, India among older adults more than or equal to 60 years of age selected using two stage, simple random sampling. The data was collected in a sample of 400 older adults by face-to-face interview using self-designed, semi-structured and pretested proforma that included validated Hindi version of Geriatric Depression Scale (GDS-15) and analyzed using SPSS v23.

**Results:**

The prevalence of depression among older adults was 51.5% in the present study; with 27%, 12.8% and 11.8% having mild, moderate and severe depression respectively. Number of family members, living status of spouse, emotional attachment to family members, conflict in family, loneliness, social isolation, marginal friendship ties, functional status, physical exercise, active complains and diastolic BP were independent predictors of depression in older adults.

**Conclusion:**

Early identification of depression in older adults using GDS-15 and incorporation of social isolation and functionality assessment routinely by healthcare providers for all older adults attending the outpatient departments is the need of the hour. A holistic approach to care of older adults is vital as healthcare providers seek to understand the impact of multiple, complex, interconnected factors on overall health and well-being of older adults.

**Supplementary Information:**

The online version contains supplementary material available at 10.1186/s12877-023-04402-2.

## Key points

1. More than half the older adults in urban slums of India are depressed.

2. Number of family members, living status of spouse, emotional attachment to family members, conflict in family, loneliness, social isolation, marginal friendship ties, functional status, physical exercise, active complains and diastolic BP were independent predictors of depression in older adults.

3. Early identification of depression in older adults using GDS-15 and incorporation of social isolation and functionality assessment routinely by healthcare providers for all older adults attending the outpatient departments is the need of the hour.

4. Why Does This Paper Matter?

The study has identified the necessity of having a competent system at primary healthcare delivery points for early identification and management of depression in older adults. This is an addition to incorporating social isolation and functionality assessment routinely by healthcare providers for all older adults attending the outpatient departments.

**Supplementary Information:**

The online version contains supplementary material available at 10.1186/s12877-023-04402-2.

## Impact statement

We certify that this work is novel. Firstly, though studies have been conducted in rural and urban populations across India on prevalence of depression in older adults; showing rural older adults with increased risk of depression in comparison to urban older adults, the vulnerability of older adults in urban slums of India is relatively less explored. Secondly, we could identify factors that significantly predict depression in older adults; among sociodemographic factors, family support factors, psychosocial factors, lifestyle factors, and medical factors, in addition to overall predictors of depression in older adults. Also, the study has identified the necessity of having a competent system at primary healthcare delivery points across the country for early identification and management of depression in older adults. This is an addition to incorporating social isolation and functionality assessment routinely by healthcare providers for all older adults attending the outpatient departments.

## Introduction

The world’s population is ageing rapidly [1]. Globally, compared to 2017, the number of persons aged 60 or above is expected to more than double by 2050 and to more than triple by 2100, rising from 962 million in 2017 to 2.1 billion in 2050 and 3.1 billion in 2100 [2]. This can be attributed to dramatic increase in life expectancy due to major advances in medicine and technology, increased focus on public health, improved education, and better nutrition [3]. India is no different. Epidemiological and demographic transition coupled with improvements in health has resulted in a steady increase in proportion of Indian older adults from 5.3% in 1951 to 8.6% in 2011 [4]; expected to increase to 12.7% by 2026 [5, 6].

It is not only the total number of older adults, but also the number of older adults in urban areas that is steeply increasing [7]. Globally, the proportion of older adults population is growing faster in urban areas than in rural areas in all the regions except Oceania [2]. In Asia, compared to 2000, the number of older adults in urban areas more than doubled in 2015, while in rural areas the number of older adults increased by just 28% over the same period [8]. In India, the share of older adults in total urban population has increased from 4.7% in 1961 to 8.1% in 2011 [5, 9]. The rise of slum as a consequence of rapid urbanization has resulted in its own set of problems [10, 11]. In 2011, 17.4% of urban Indian households lived in a slum. Nearly one in every six urban Indian residents lives in a slum. More than one in five urban households in Chhattisgarh lives in a slum [12, 13].

Mental health is an integral part of overall health and well-being (SDG 3) [14]. Worldwide, over 20% of older adults suffer from a mental or neurological disorder (excluding headache disorders) [15]; attributes to 6.6% of total Disability Adjusted Life Years (DALYs) and 17.4% of Years Lived with Disability (YLDs) [16, 17]. The estimated economic consequences of mental disorders in terms of lost economic output amounts to US$ 16.3 million between 2011 and 2030 [18]. Approximately 7% of the world’s older adult population is affected by depression, the most common mental and neurological disorder – single largest contributor to non-fatal health loss (5.7% of YLDs) [15, 19, 20].

India is home to nearly 104 million older adults and this number is likely to increase [9], with half of this population suffering from some chronic disease and more than 10.9% requiring mental health care [21]. Older adults are vulnerable to chronic and silent depressive changes which can be enhanced by social neglect, physical and economical dependability, loss of spouse or near ones, increased disease susceptibility and chronic debility. The perception of poor health, utilization of healthcare services and costs are also increased with depression [22].

There are major gaps in identifying cases (burden of disease) and understanding depression in older adults population (factors influencing depression). Existing evidence shows that rural older adults are at increased risk of depression in comparison to urban older adults [23]. However, the vulnerability of older adults in urban slums of India is relatively less explored. Against this background, the objective of this study was to estimate the prevalence of depression among the older adults (≥ 60 years) in the Urban Field Practice Area of AIIMS Raipur and to study the factors associated with it.

### Subjects and methods

This was a community based analytical cross-sectional study carried out in Urban Field Practice Area (UFPA) (Wards 18 and 19 of Zone 8) of Department of Community and Family Medicine, All India Institute of Medical Sciences (AIIMS), Raipur, India between May 2020, and July 2020. The study enrolled older adults (≥ 60 years) residing in the study area for at least six months preceding the date of survey (permanent residents) and having Hindi Mental Status Examination (HMSE) scale scores > 19 (amongst illiterate) or > 24 (amongst literate) [24]. Older adults people not at home even after the third visit, debilitated, with hearing impairment and blindness, with history of psychosis, bipolar depression, schizophrenia, and use of psychotropic drugs were excluded [25].

In a meta-analysis conducted by Barua A et al., the median prevalence rate of depression among Indian older adults was 21.9% (interquartile range (IQR) 11.6 to 31.1) [26]. Taking the prevalence to be 11.6% and applying 5% absolute error, considering 20% non-response and design effect of 2 – the minimum required sample size was rounded to be 400 with 95% confidence (CI).

A representative sample of the study population was drawn from the study area using two stage, simple random sampling. The UFPA covers Zone 8 (17 areas, 4273 houses) which included Ward 18 (10 areas, 3241 houses) and Ward 19 (7 areas, 1032 houses). Considering equal distribution of houses in areas of Ward 18 (300 to 350 houses) and areas of Ward 19 (120 to 160 houses); out of 400 samples to be collected, 320 (80%) samples were taken from Ward 18 and 80 (20%) were taken from Ward 19 (Ratio of 4:1). Then 6 areas were selected randomly, 4 from Ward 18 and 2 from Ward 19 by lottery method as shown in Additional file 1: Appendix 1. A street was selected randomly in each area after which the first house was selected by rotating water bottle. The first house was randomly selected in the direction of the tip of the water bottle and houses were further visited consecutively in the right direction. In the case of multi-floor, multiple dwelling units, the order of enumeration of houses was from bottom to top. In houses with two or more older adults, the eldest of them fulfilling inclusion and exclusion criteria were enrolled in the study. The data was collected by face-to-face interview using self-designed, semi-structured and pretested proforma that included – socio-demographic profile, family support, psychosocial, and lifestyle factors. The medical factors included self-reported active complaints; chronic conditions and polypharmacy obtained from medical records followed by self-reported that in order of preference; polypharmacy [27]; anemia/pallor (by examination); and systolic and diastolic pressure (two measurements separated by 1–2 min and an average taken) [28]. The primary outcome or dependent variable ‘depression in older adults’ was obtained using validated Hindi version of Geriatric Depression Scale (GDS-15) – an older adult with scores less than 5 was considered not to have depression, whereas scores 5 to 8, 9 to 11 and 12 to 15 suggested mild, moderate, and severe depression respectively. GDS-15 has a sensitivity of 92% and a specificity of 81% with a high degree of internal consistency, for identifying depression among older adults [29]. Other scales used in the study included Lubben Social Network Scale – 6 (LSNS-6), a 6-item questionnaire to assess perceived social support received from family and friends that has demonstrated high levels of internal consistency (0.83), stable factor structures, and high correlations with criterion variables among older population; and Katz Activities of Daily Living (Katz ADL) index, a 6-item questionnaire to assess functional status by measuring the participants ability to perform activities of daily living independently [30–33].

Data was collected from 400 participants, entered in Microsoft Excel, and analyzed using Statistical Package for the Social Sciences (SPSS) v23. Descriptive analysis was presented using numbers and percentages. The Chi square test of significance (two-sided) was applied to test for association between independent variables and depression; independent study variables were not associated with depression was the null hypothesis (H_0_) and presence of association between independent variables and depression was the alternate hypothesis (H_1_). Univariate odds ratio was estimated along with 95% CI for these variables. All predictor variables significant at *p* < 0.05 in univariate analysis were included in multiple logistic regression analysis using pre-final and final model. The goodness of fit of the final model was evaluated.

The study was approved by Institute Ethics Committee, All India Institute of Medical Sciences, Raipur, Chhattisgarh, India (AIIMSRPR/IEC/2018/178). The content of Participant Information Sheet (PIS) in local language was provided to the participants and contents were read to them in their own language to their satisfaction. The participants were enrolled in the study after obtaining written informed consent.

## Results

The distribution of participants according to socio-demographic factors is illustrated in Additional file 1: Appendix 2. Out of total 400 participants, maximum 176 (44.0%) were found to be in the age group of 60 to 64 years followed by 166 (41.5%) in the age group of 65 to 74 years. Enrollment was found to be higher for females 231 (57.8%) as compared to males 169 (42.3%). Majority, 390 (97.5%) participants belonged to Hindu religion. Almost two thirds, 260 (65%) participants were illiterate and more than one fourth, 109 (27.2%) participants reported to be working. Extended family was the common family type accounting for 272 (68%) families and only 18 (4.5%) families were of joint type. Maximum, 270 (67.5%) participants belonged to upper lower socio-economic class followed by 88 (22%) to lower socioeconomic class. Almost half, 191 (47.8%) participants were a part of family with more than or equal to 6 members whereas only 32 (8%) were a part of family with less than three study subjects. Of the older adults with their spouse alive, 1.6% were living alone due to divorce or separation; and of the older adults with their spouse dead, 13.9% were living alone.

### Prevalence of depression

The prevalence of depression among older adults was 51.5% in the present study; with 27%, 12.8% and 11.8% having mild, moderate, and severe depression respectively. Of those with mild, moderate, and severe depression 55.6%, 56.9% and 66% were females respectively as shown in Additional file 1: Appendix 3. Females contributed to 58.3% of observed depression prevalence whereas males contributed to 41.7% of depression.

### Factors associated with depression in older adults

Outline of the analysis done, and models used are illustrated in Additional file 1: Appendix 4. Also, the flow of factors in relation to basic, pre-final and final models are shown in Additional file 1: Appendix 5. In the pre-final model, when adjusted for other variables in the respective groups; lower socioeconomic status, less than three family members, dead spouse financial dependance (demographic factors, Table 1); absence of emotional attachment with family members, presence of conflict in the family (family support factors, Table 2); feeling lonely, being socially isolated, presence of marginal friendship ties, being not fully functional (psychosocial factors, Table 3); lack of physical exercise, spiritual inactivity, lack of sound sleep (lifestyle factors, Table 4); presence of active complaints, systolic BP more than or equal to 150, diastolic BP more than or equal to 90, presence of pallor (medical factors, Table 5) showed significant association with depression in older adults.

**Table 1:** Association between socio-demographic factors and depression

**Socio-demographic factors**		**Depression**				**Total**		**Basic model**	**Pre-final model**	**Final model**
		**Present (*n* = 206)**		**Absent (*n* = 194)**				**OR (95% CI)**	**AOR (95% CI)**	**AOR (95% CI)**
		**No**	**%**	**No**	**%**	**No**	**%**			
**Age**^**a**^	**60–64**	84	40.8	92	47.4	176	44.0	1	-	-
	**65–74**	89	43.2	77	39.7	166	41.5	1.27 (0.83–1.94)	-	-
	** > 75**	33	16.0	25	12.9	58	14.5	1.45 (0.79–2.63)	-	-
**Sex**	**Male**	86	41.7	83	42.8	169	42.3	1	-	-
	**Female**	120	58.3	111	57.2	231	57.8	1.04 (0.70–1.55)	-	-
**Type of family**^**b**^	**Nuclear**	76	36.9	34	17.5	110	27.5	2.75 (1.73–4.38)	1.07 (0.51–2.26)	-
	**Extended/ Joint**	130	63.1	160	82.5	290	72.5	1	1	-
**Number of family members**	** < 3**	67	32.5	19	9.8	86	21.5	4.99 (2.79–8.98)	4.23 (1.74–10.29)	3.68 (1.17–11.57)
	**3–5**	60	29.1	63	32.5	123	30.8	1.35 (0.86–2.13)	1.35 (0.82–2.21)	0.92 (0.33–2.51)
	** > 6**	79	38.4	112	57.7	191	47.8	1	1	1
**Education**	**Literate**	65	31.6	75	38.7	140	35.0	1	-	-
	**Illiterate**	141	68.4	119	61.3	260	65.0	1.37 (0.91–2.06)	-	-
**Employment**	**Present**	51	24.8	58	29.9	109	27.3	1	-	-
	**Absent**	155	75.2	136	70.1	291	72.8	1.30 (0.84–2.02)	-	-
**Socio-economic Status**^**c**^	**Upper and middle**	10	4.9	32	16.5	42	10.5	1	1	1
	**Lower**	196	95.1	162	83.5	358	89.5	3.87 (1.85–8.11)	3.03 (1.40–6.58)	4.39 (0.84–22.84)
**Marital status**	**Married**	205	99.5	194	100	399	99.8	-	-	-
	**Unmarried**	1	0.5	0	0.0	1	0.3	-	-	-
**Living status of spouse (*n* = 399)**	**Alive**	87	42.4	103	53.1	190	47.7	1	1	1
	**Dead**	118	57.6	91	46.9	209	52.3	1.54 (1.03–2.28)	1.65 (1.06–2.58)	3.39 (1.29–8.93)
**Number of children (*n* = 399)**	**No children**	10	4.9	2	1.0	12	3.0	5.41 (1.16–25.13)	2.80 (0.54–14.61)	-
	**1–2 children**	60	29.3	46	23.7	106	26.6	1.41 (0.90–2.21)	1.24 (0.75–2.03)	-
	** > 3 children**	135	65.8	146	75.3	281	70.4	1	1	-
**Financial dependence**	**Present**	135	65.5	97	50.0	232	58.0	1.90 (1.27–2.84)	1.94 (1.25–3.01)	0.57 (0.22–1.48)
	**Absent**	71	34.5	97	50.0	168	42.0	1	1	1

^a^Participants between the ages of 60 and 64 years were identified as youngest-old, those between ages 65 and 74 years as middle-old, and those aged 75 years and over as oldest-old

^b^The type of family was categorized into nuclear (married couple with or without minor/dependent/adopted children), joint (two or more blood related individuals (usually brothers) and their spouses, with or without their children) and extended (vertical extension of the nuclear family, with representation of three generations where a married couple along with their minor/dependent children lived with the older adults parents) family

^c^Socioeconomic status was based on Modified Kuppuswamy scale updated for the year 2018 (I, upper; II, upper middle; III, lower middle; IV, upper lower; V, lower)

**Table 2:** Association between family support factors and depression

**Family support factors**		**Depression**				**Total**		**Basic model**	**Pre-final model**	**Final model**
		**Present (*n* = 206)**		**Absent (*n* = 194)**				**OR (95% CI)**	**AOR (95% CI)**	**AOR (95% CI)**
		**No**	**%**	**No**	**%**	**No**	**%**			
**Spends sufficient time with children in the family**	**Yes**	143	69.4	187	96.4	330	82.5	1	1	-
	**No**	63	30.6	7	3.6	70	17.5	11.77 (5.23–26.47)	2.94 (0.83–10.37)	-
**Family members initiating conversation**	**Yes**	149	72.3	190	97.9	339	84.8	1	1	-
	**No**	57	27.7	4	2.1	61	15.3	18.17 (6.45–51.22)	3.09 (0.59–16.09)	-
**Emotionally attached with family members**	**Yes**	137	66.5	187	96.4	324	81.0	1	1	1
	**No**	69	33.5	7	3.6	76	19.0	13.46 (6.00–30.19)	8.01 (2.70–23.75)	4.40 (1.04–18.56)
**Presence of maximum support in the family**	**Yes**	154	74.8	187	96.4	341	85.3	1	1	-
	**No**	52	25.2	7	3.6	59	14.8	9.02 (3.98–20.43)	0.61 (0.16–2.35)	-
**Presence of conflict in the family**	**Yes**	46	22.3	5	2.6	51	12.8	10.87 (4.22–28.01)	11.49 (4.34–30.43)	26.62 (4.68–151.39)
	**No**	160	77.7	189	97.4	349	87.2	1	1	1

**Table 3:** Association between psychosocial factors and depression

**Psychosocial factors**		**Depression**				**Total**		**Basic model**	**Pre-final model**	**Final model**
		**Present (*n* = 206)**		**Absent (*n* = 194)**				**OR (95% CI)**	**AOR (95% CI)**	**AOR (95% CI)**
		**No**	**%**	**No**	**%**	**No**	**%**			
**Visiting friend/group/ place on regular basis**	**Yes**	99	48.1	175	90.2	274	68.5	1	1	-
	**No**	107	51.9	19	9.8	126	31.5	9.96 (5.76–17.20)	0.56 (0.19–1.66)	-
**Attending social functions**	**Yes**	134	65.0	192	99.0	326	81.5	1	1	-
	**No**	72	35.0	2	1.0	74	18.5	51.58 (12.44–213.88)	2.13 (0.35–12.94)	-
**Stressful life event in the past one year**	**Yes**	87	42.2	18	9.3	105	26.3	7.15 (4.09–12.49)	1.62 (0.64–4.14)	-
	**No**	119	57.8	176	90.7	295	73.8	1	1	-
**Health status perceived as poor**	**Yes**	152	73.8	41	21.1	193	48.3	10.50 (6.61–16.70)	1.96 (0.86–4.47)	-
	**No**	54	26.2	153	78.9	207	51.8	1	1	-
**Feeling lonely**	**Yes**	143	69.4	20	10.3	163	40.8	19.75 (11.40–34.21)	4.09 (1.79–9.31)	2.85 (1.07–7.56)
	**No**	63	30.6	174	89.7	237	59.3	1	1	1
**Social isolation**^**a**^	**Present**	135	65.5	2	1.0	137	34.3	182.54 (44.01–757.01)	16.20 (1.78–147.38)	11.87 (1.99–70.70)
	**Absent**	71	34.5	192	99.0	263	65.8	1	1	1
**Marginal family ties**^**b**^	**Present**	58	28.2	1	0.5	59	14.8	75.64 (10.36–552.40)	1.10 (0.06–21.79)	-
	**Absent**	148	71.8	193	99.5	341	85.3	1	1	-
**Marginal friendship ties**^**c**^	**Present**	166	80.6	8	4.1	174	43.5	96.49 (43.90–212.05)	12.14 (4.43–33.29)	17.59 (5.17–59.55)
	**Absent**	40	19.4	186	95.9	226	56.5	1	1	1
**Functionality**^**d**^	**Not fully functional**	27	13.1	1	0.5	28	7.0	29.11 (3.92–216.46)	17.90 (1.58–202.94)	35.48 (1.97–638.34)
	**Fully functional**	179	86.9	193	99.5	372	93.0	1	1	1

^a^A person with total LSNS-6 score less than 12 was said to have social isolation

^b^A person with LSNS-6 family score less than 6 was said to have marginal family ties

^c^A person with LSNS-6 friendship score less than 6 was said to have marginal friendship ties

^d^Based on Katz index Activities of Daily Living (Katz ADL), score of 6 was considered as the person having no functional impairment. A score of 3 to 5 was considered moderate functional impairment whereas 2 or less was considered to have severe functional impairment

**Table 4:** Association between lifestyle factors and depression

**Lifestyle factors**		**Depression**				**Total**		**Basic model**	**Pre-final model**	**Final model**
		**Present (*n* = 206)**		**Absent (*n* = 194)**				**OR (95% CI)**	**AOR (95% CI)**	**AOR (95% CI)**
		**No**	**%**	**No**	**%**	**No**	**%**			
**Physical exercise**	**Yes**	11	5.3	45	23.2	56	14.0	1	1	1
	**No**	195	94.7	149	76.8	344	86.0	5.35 (2.68–10.71)	4.13 (1.87–9.12)	13.77 (3.18–59.70)
**Practice of yoga/ meditation**	**Yes**	11	5.3	19	9.8	30	7.5	1	-	-
	**No**	195	94.7	175	90.2	370	92.5	1.93 (0.89–4.16)	-	-
**Spiritual activity**	**Yes**	152	73.8	186	95.9	338	84.5	1	1	1
	**No**	54	26.2	8	4.1	62	15.5	8.26 (3.81–17.89)	5.85 (2.56–13.38)	2.66 (0.68–10.38)
**Sound sleep**	**Yes**	126	61.2	187	96.4	313	78.3	1	1	1
	**No**	80	38.8	7	3.6	87	21.8	16.96 (7.58–37.93)	14.54 (6.33–33.41)	4.71 (0.92–24.06)
**Smoking**	**Yes**	35	17.0	23	11.9	58	14.5	1.52 (0.86–2.68)	-	-
	**No**	171	83.0	171	88.1	342	85.5	1	-	-
**Smokeless tobacco use**	**Present**	126	61.2	91	46.9	217	54.2	1.78 (1.20–2.65)	1.54 (0.96–2.46)	-
	**Absent**	80	38.8	103	53.1	183	45.8	1	1	-
**Alcohol use**	**Yes**	33	16.0	30	15.5	63	15.8	1.04 (0.61–1.79)	-	-
	**No**	173	84.0	164	84.5	337	84.3	1	-	-

**Table 5:** Association between medical factors and depression

**Medical factors**		**Depression**				**Total**		**Basic model**	**Pre-final model**	**Final model**
		**Present (*n* = 206)**		**Absent (*n* = 194)**				**OR (95% CI)**	**AOR (95% CI)**	**AOR (95% CI)**
		**No**	**%**	**No**	**%**	**No**	**%**			
**Active complaints**	**Present**	78	37.9	37	19.1	115	28.8	2.59 (1.64–4.08)	2.52 (1.54–4.10)	2.70 (1.03–7.08)
	**Absent**	128	62.1	157	80.9	285	71.3	1	1	1
**Chronic condition**	**Present**	89	43.2	69	35.6	158	39.5	1.38 (0.92–2.06)	-	-
	**Absent**	117	56.8	125	64.4	242	60.5	1	-	-
**Polypharmacy**	**Present**	61	29.6	38	19.6	99	24.8	1.73 (1.09–2.75)	1.02 (0.60–1.73)	-
	**Absent**	145	70.4	156	80.4	301	75.3	1	1	-
**SBP**	** < 150**	186	90.3	191	98.5	377	94.2	1	1	1
	** > = 150**	20	9.7	3	1.5	23	5.8	6.85 (2.00–23.43)	3.98 (1.08–14.71)	0.90 (0.11–7.82)
**DBP**	** < 90**	125	60.7	160	82.5	285	71.2	1	1	1
	** > = 90**	81	39.3	34	17.5	115	28.8	3.05 (1.92–4.85)	2.20 (1.31–3.70)	3.33 (1.23–8.99)
**Anemia (Pallor)**	**Present**	159	77.2	108	55.7	267	66.7	2.69 (1.75–4.15)	2.35 (1.49–3.69)	1.33 (0.54–3.27)
	**Absent**	47	22.8	86	44.3	133	33.3	1	1	1

In the final model, when adjusted for other variables across the groups, less than three family members (AOR 3.68, 95%CI 1.17 to 11.57), dead spouse (AOR 3.39, 95%CI 1.29 to 8.93), absence of emotional attachment with family members (AOR 4.40, 95%CI 1.04 to 18.56), presence of conflict in the family (AOR 26.62, 95%CI 4.68 to 151.39), feeling lonely (AOR 2.85, 95%CI 1.07 to 7.56), being socially isolated (AOR 11.87, 95%CI 1.99 to 70.70), presence of marginal friendship ties (AOR 17.59, 95%CI 5.17 to 59.55), being not fully functional (AOR 35.48, 95%CI 1.97 to 638.34), lack of physical exercise (AOR 13.77, 95%CI 3.18 to 59.70), presence of active complaints (AOR 2.70, 95%CI 1.03 to 7.08) and diastolic BP more than or equal to 90 (AOR 3.33, 95%CI 1.23 to 8.99) emerged as independent predictors of depression in older adults.

The goodness of fit of the final model was judged by Hosmer and Lemeshow test which was not significant (*p* = 0.938), and the Omnibus test of model coefficient was significant (*p* =  < 0.001) – indicating a good fit model. The model was able to predict 92% of the outcome, which was much better than the null hypothesis (51.4%). The final model can explain the variance in outcome variable, that is, depression in older adults by 83.2% (Nagelkerke R^2^ = 0.832).

## Discussion

The present study findings showed that every other older adult residing in an urban slum of Raipur city was depressed. A retrospective study determined global median prevalence rate of depressive disorders for the older population to be 10.3% (IQR 4.7 to 16.0) using community-based cross-sectional surveys and prospective studies [26]. In a recent systematic review that included studies between 1994 and 2020 conducted globally, the prevalence of depression ranged from 7.7% to 81.1%; and the average prevalence of depression among old age was 31.7% (95%CI 27.9 to 35.6) [34]. In a meta-analysis published in 2019 which included fifty-one studies from 16 States of India, the estimated prevalence of depression among Indian older adults population was 34.4% (95%CI 29.3 to 39.7) [23]. Urban slum population of lower socioeconomic status (SES) and higher sensitivity of study tool may have resulted in a higher prevalence of depression in this study. Despite recognition of the burden of disease due to depression and other mood disorders in low- and middle-income countries (LMICs), depression in primary care settings is underdetected, underdiagnosed, and undertreated [35]. The absolute difference between the prevalence of mental disorders and the proportion treated; treatment gap has been found to be 76% to 85% in less-developed countries [36]. The National Mental Health Survey (NMHS) of India, 2016 reports an overall treatment gap of 83% for any mental health problem – 85.0% for common mental disorders, and 73.6% for severe mental disorders [37, 38]. The barriers include stigma surrounding these conditions making people reluctant to seek help, inadequate appreciation of the inter-play between mental illness and other health disorders, existing public-health priorities, and its influence on funding [39]. Additionally, India has two mental health workers and 0.3 psychiatrists per 100,000 population; much lower than the global average. Addressing the unmet needs of mental health-care delivery in India necessitates innovative and novel methods [21, 40].

Older adults with less than three family members, dead spouse, absence of emotional attachment with family members, presence of conflict in the family, feeling lonely, being socially isolated, presence of marginal friendship ties, not fully functional, lack of physical exercise, presence of active complaints and diastolic BP more than or equal to 90 were at increased risk of depression in the present study.

Existing studies highlight that joint and extended families provided more emotional and monetary support to the older adults. The relative contraction of these family types and simultaneous emergence of the nuclear family, with consequent smaller family size may explain the higher occurrence of depression in recent years [41]. Every human being is an individual embodiment of social relations and the tendency to withdraw from the society in the form of not visiting friend or group or place on regular basis and not attending social functions may result in depression [42]. Similarly, marginal friendship ties were a better indicator of social isolation than marginal family ties in this study. However, socioemotional selectivity theory states that aging causes an intentional social network pruning to procure and maintain emotionally fulfilling bonds, while shedding weaker, less supportive relationships [43]. Though the association between social networks, depression and functionality is certain, evidence shows that causality may be attributed to any of the three, in relation to others [43].

Physical exercise is known to be as effective as drugs to prevent depression. The possible mechanisms for the beneficial effects may be thermogenic, endorphins, monoamine, distraction, or self-efficacy hypothesis. However, a combination of biological, psychological, and sociological factors is likely to influence the relationship between exercise and depression [44]. In the present study, the findings support the notion that spirituality is beneficial against depression. The mechanisms through which potential benefits may occur are coping styles, locus of control, social support, social networks, physiological mechanisms, and architecture & the built environment [45]. It is known fact that unsound sleep is common in depressed older adults and may be the presenting complaint. Depressed individuals may suffer from a range of insomnia symptoms, including difficulty falling asleep (sleep onset insomnia), difficulty staying asleep (sleep maintenance insomnia), un-refreshing sleep, and daytime sleepiness. However, the risk of developing depression is highest among people with both sleep onset and sleep maintenance insomnia [46]. The relationship between hypertension and depression may be due to increased adrenergic activity in hypertension leading to depression among older adults. The other possible explanation could be that somatic symptoms, lower quality of life, lack of occupational and social role function associated with hypertension may predispose to psychological distress in older adults, especially depression [47]. The ease of using these factors in community or as a part of Comprehensive Geriatric Assessment (CGA) in healthcare settings presents an opportunity for early identification of older adults at risk of depression.

This study draws evidence on a representative population of urban slum older adults to report on the prevalence of depression applicable to older adults from similar other settings. However, there are a few limitations. First, the selection of eldest in houses with more than one older person may have introduced systematic bias in relation to age distribution of the sample. However, the age was normally distributed in the sample (Shapiro–Wilk Test, > 0.05). Second, the length of proforma restricted using an objective sleep quality questionnaire. Third, the temporality of predictors of depression cannot be established (inherent limitation of study design). Fourth, the family support variables could have been qualitative; however, the rapport and familiarity with the study area and participants meant that the categorical (Yes/No) data capture would be reliable and accurate.

The study findings have important implications. A competent system should be in place at the Urban Health Training Centre (UHTC) of AIIMS Raipur and similar other primary healthcare delivery points across the country for early identification and management of depression in older adults. Incorporating social isolation and functionality assessment along with use of GDS-15 routinely by healthcare providers for all older adults attending the outpatient departments should be considered. All family members should be sensitized about psychosocial support given to older adults in their families as a part of home health care for older adults. This can be supported by formation of self-help groups and engagement of older adults in community-based activities of UHTC, AIIMS, Raipur or other primary healthcare delivery points. The scope of using GDS-15 for routine community screening of older adults in addition to risk assessment based on predictors of this study should be explored.

To conclude, a holistic approach to care of older adults is vital as healthcare providers seek to understand the impact of multiple, complex, interconnected physical, mental, spiritual, and social factors on overall health and well-being of older adults.

### Supplementary Information

**Additional file 1: Appendix 1. **Study universe with method of sample selection. **Appendix 2.** Distribution of study subjects according to socio-demographic factors. **Appendix 3.** Distribution of depression in elderly according to sex. **Appendix 4.** Outline of the analysis done, and models used. **Appendix 5.** The flow of factors in relation to basic, pre-final and final models.

## Data Availability

The datasets used and/or analyzed during the current study available from the corresponding author on reasonable request.
