# Supplementary material for: Depression among older adults in an urban slum of Raipur city – a community based cross-sectional study
Source: BMC Geriatr. 2023 Nov 2;23:709. doi: 10.1186/s12877-023-04402-2 (PMC10621310; doi:10.1186/s12877-023-04402-2)
Supplement: Supplementary file 1 — Additional file 1: Appendix 1. Study universe with method of sample selection. Appendix 2. Distribution of study subjects according to socio-demographic factors. Appendix 3. Distribution of depression in elderly according to sex. Appendix 4. Outline of the analysis done, and models used. Appendix 5. The flow of factors in relation to basic, pre-final and final models. [file 12877_2023_4402_MOESM1_ESM.docx]

**Supplementary file 1**

**Appendix 1.** Study universe with method of sample selection
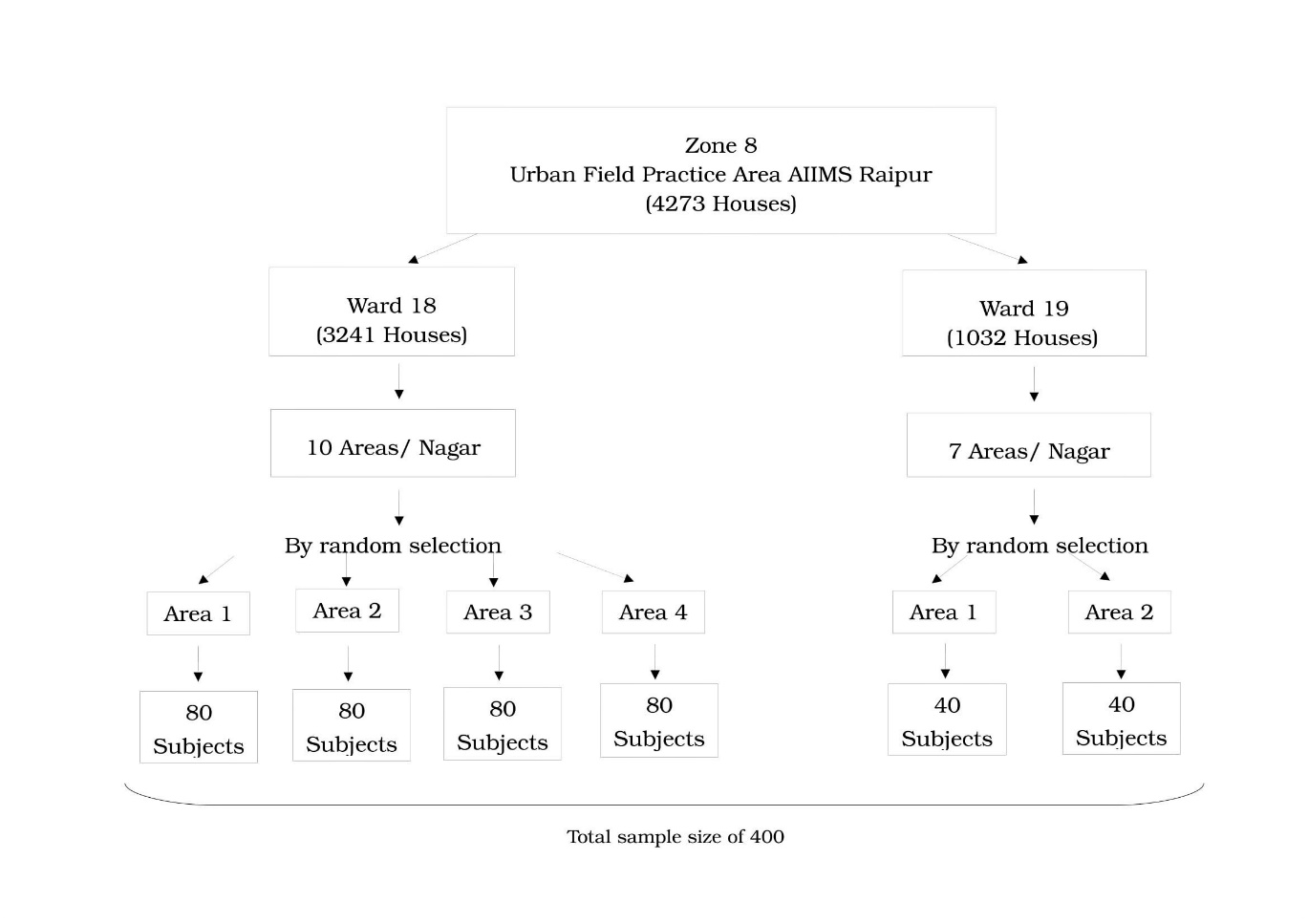


**Appendix 2.** Distribution of study subjects according to socio-demographic factors

| **Socio-demographic factors** | | **Study subjects** | |
| --- | --- | --- | --- |
|  |  | **No.** | **%** |
| **Age (in years)** | **60-64** | 176 | 44.0 |
|  | **65-74** | 166 | 41.5 |
|  | **>75** | 58 | 14.5 |
| **Sex** | **Male** | 169 | 42.3 |
|  | **Female** | 231 | 57.8 |
| **Religion** | **Hindu** | 390 | 97.5 |
|  | **Non-Hindu** | 10 | 2.5 |
| **Type of family** | **Nuclear** | 110 | 27.5 |
|  | **Extended** | 272 | 68.0 |
|  | **Joint** | 18 | 4.5 |
| **Number of family members** | **<3** | 86 | 21.5 |
|  | **3-5** | 123 | 30.8 |
|  | **>6** | 191 | 47.8 |
| **Education** | **Literate** | 140 | 35.0 |
|  | **Illiterate** | 260 | 65.0 |
| **Employment** | **Present** | 109 | 27.3 |
|  | **Absent** | 291 | 72.8 |
| **Socio-Economic Status** | **Upper** | 2 | 0.5 |
|  | **Upper middle** | 11 | 2.8 |
|  | **Lower middle** | 29 | 7.3 |
|  | **Upper lower** | 270 | 67.5 |
|  | **Lower** | 88 | 22.0 |
| **Marital status** | **Married** | 399 | 99.8 |
|  | **Unmarried** | 1 | 0.3 |

**Appendix 3:** Distribution of depression in elderly according to sex

**Appendix 4:** Outline of the analysis done, and models used

- All the variables were cross tabulated with presence or absence of depression;
- Test of significance ‘Chi square’ was calculated;
- Odds Ratio (OR) along with 95% CI and p Value were calculated;
- 29 variables were significantly associated with depression.

**Basic model**

- 29 variables were included in the pre-final model;
- These variables were grouped into demographic (6 variables), family support (5 variables), psychosocial (9 variables), lifestyle (4 variables) and medical (5 variables) factors;
- Multiple Logistic Regression applied for each group (adjusted for other variables in the same group) and AOR was calculated;
- 17 variables were found significant.

**Pre-final model**

- 17 significant variables of different groups were included;
- Multiple Logistic Regression applied (adjusted for other variables across the groups) and AOR was calculated;
- Out of 17 variables, 11 were found significant in the final model.

**Final model**

**Appendix 5:** The flow of factors in relation to basic, pre-final and final models.

| **Variables in the study** | **Variables found significant in basic model** | **Variables found significant in pre-final model** | **Variables found significant in final model** |
| --- | --- | --- | --- |
| **Demographic variables (11):**   1. Age 2. Sex 3. Type of family 4. Number of family members 5. Education 6. Employment 7. Socio-economic status 8. Marital status 9. Living status of spouse 10. Number of children 11. Financial dependence   **Family support (5):**   1. Spends sufficient time with children in the family 2. Family members initiating conversation 3. Emotionally attached with family members 4. Presence of maximum support in the family 5. Presence of conflict in the family   **Psychosocial factors (9):**   1. Visiting friend/group/place on regular basis 2. Attending social functions 3. Stressful life event in the past one year 4. Health status perceived as poor 5. Feeling lonely 6. Social isolation (LSNS) 7. Marginal family ties 8. Marginal friendship ties 9. Functionality   **Lifestyle factors (7):**   1. Physical exercise 2. Practice of yoga/meditation 3. Spiritual activity 4. Sound sleep 5. Smoking 6. Smokeless tobacco use 7. Alcohol use   **Medical history (6):**   1. Active complaints 2. Chronic condition 3. Polypharmacy 4. Systolic BP 5. Diastolic BP 6. Anemia (Pallor) | **Demographic variables (6):**   1. Type of family 2. Socio-economic status 3. Number of family members 4. Living status of spouse 5. Number of children 6. Financial dependence   **Family support (5):**   1. Spends sufficient time with children in the family 2. Family members initiating conversation 3. Emotionally attached with family members 4. Presence of maximum support in the family 5. Presence of conflict in the family   **Psychosocial factors (9):**   1. Visiting friend/group/place on regular basis 2. Attending social functions 3. Stressful life event in the past one year 4. Health status perceived as poor 5. Feeling lonely 6. Social isolation (LSNS) 7. Marginal family ties 8. Marginal friendship ties 9. Functionality   **Lifestyle factors (4):**   1. Physical exercise 2. Spiritual activity 3. Sound sleep 4. Smokeless tobacco use   **Medical history (5):**   1. Active complaints 2. Polypharmacy 3. Systolic BP 4. Diastolic BP 5. Anemia (Pallor) | **Demographic variables (4):**   1. Socio-economic status 2. Number of family members 3. Living status of spouse 4. Financial dependence   **Family support (2):**   1. Emotionally attached with family members 2. Presence of conflict in the family   **Psychosocial factors (4):**   1. Feeling lonely 2. Social isolation (LSNS) 3. Marginal friendship ties 4. Functionality   **Lifestyle factors (3):**   1. Physical exercise 2. Spiritual activity 3. Sound sleep   **Medical history (4):**   1. Active complaints 2. Systolic BP 3. Diastolic BP 4. Anemia (Pallor) | **Demographic variables (2):**   1. Number of family members 2. Living status of spouse   **Family support (2):**   1. Emotionally attached with family members 2. Presence of conflict in the family   **Psychosocial factors (4):**   1. Feeling lonely 2. Social isolation (LSNS) 3. Marginal friendship ties 4. Functionality   **Lifestyle factors (1):**   1. Physical exercise   **Medical history (2):**   1. Active complaints 2. Diastolic BP |
